# LRP-2 likely acts downstream of EGL-20/Wnt

**DOI:** 10.17912/micropub.biology.000153

**Published:** 2019-08-27

**Authors:** Paul J Minor, Paul W Sternberg

**Affiliations:** 1 Division of Biology and Biological Engineering, Caltech, Pasadena, CA 91125; 2 Department of Biology, Hopkins Marine Station of Stanford University, Pacific Grove, CA 93950

**Table 1. LRP-2 is downstream of EGL-20/Wnt:**
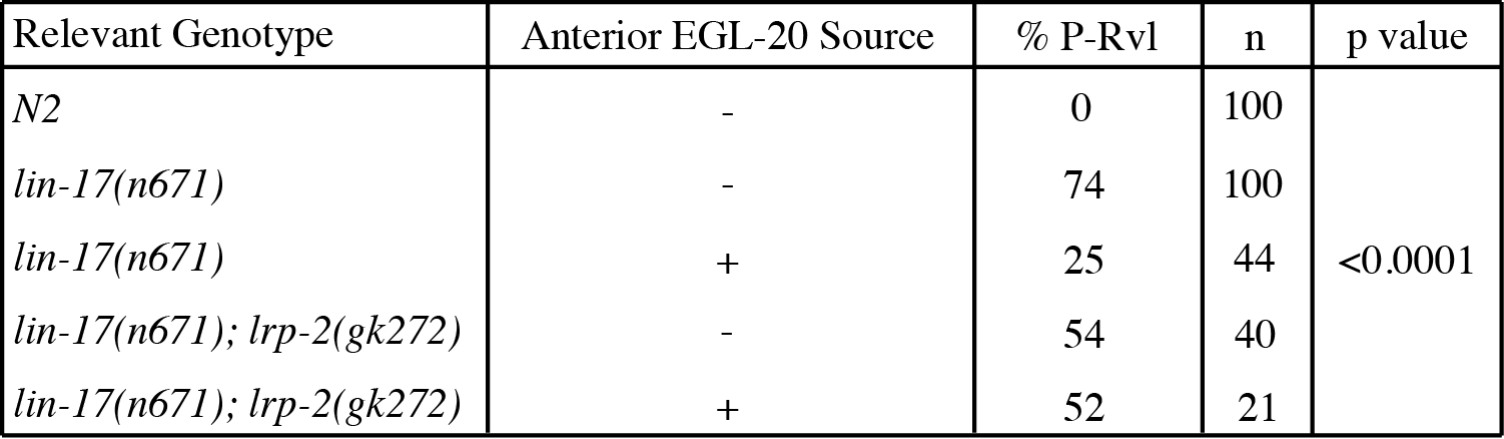
Data illustrating the proportion of posterior-reversed vulval lineage (% P-Rvl). By ectopically expressing EGL-20 from the anterior side of P7.p using the Pfos-1a promoter, P5.p and P7.p reorient to face the anterior gradient. The anterior source of EGL-20 suppresses the *lin-17*(*n671*) phenotype. Because we do not see further suppression of the *lin-17*(*n671*) *lrp-2*(*gk272*) phenotype when EGL-20 is ectopically expressed from the anterior side of P7.p we conclude that LRP-2 is downstream of EGL-20.

## Description

The *C. elegans* vulva is formed from divisions of three vulval precursor cells (VPCs) – P5.p, P6.p, and P7.p – arranged along the anteroposterior axis in the ventral epithelium (Sulston and Horvitz, 1977). Previous analyses show the orientation of P5.p and P7.p descendants is determined by the interaction of multiple Wnt signals. *egl-20*/Wnt is expressed in the tail (Whangbo and Kenyon, 2000) and forms a posterior-to-anterior concentration gradient (Coudreuse *et al*., 2006). It has previously been shown that EGL-20 acts instructively during vulva development by imparting directional information, as opposed to being permissive, where it would only be required for polarization (Green *et al*., 2008; Minor *et al*., 2013). By moving the source of *egl-20* expression from the posterior of the worm to the anchor cell, the axis of symmetry of the developing vulva, we can reorient the daughter cells of P5.p and P7.p toward the center in a wild-type configuration.

Expression of *egl-20* from the center of the axis of symmetry partially suppresses the *lin-17(n671)* phenotype (Green et al., 2008; Table 1). To test whether LRP-2 acts downstream of EGL-20, we ectopically expressed *egl-20* from the anchor cell in a *lin-17(n671); lrp-2(gk272)* double mutant background and compared it to a *lin-17(n671); lrp-2(+)* strain. If LRP-2 acts downstream of EGL-20, then anteriorly-expressed EGL-20 will not be able to suppress the *lin-17* phenotype, with is the result observed (Table 1). Thus, like CAM-1 and VANG-1, LRP-2 likely acts downstream of EGL-20.

## Reagents

**Strains:**

**VC543**: *lrp-2(gk272)*. Strain obtained from the CGC and provided by the *C. elegans* Reverse Genetics Core Facility at the University of British Columbia, which is part of the international *C. elegans* Gene Knockout Consortium.

**MT1306**: *lin-17(n671)* (Ferguson and Horvitz, 1985)

**PS5800**: *lin-17(n671); syEx1031[Pfos-1a::EGL-20::GFP]* (Green *et al*., 2008)

**MT1488**: *lin-17(n671); unc-13(e1091)*

The *lin-17(n671); lrp-2(gk272)* double mutant constructed by crossing *lrp-2(gk272)* males with strain **MT1488** hermaphrodites.

## References

[R1] Coudreuse DY, Roël G, Betist MC, Destrée O, Korswagen HC (2006). Wnt gradient formation requires retromer function in Wnt-producing cells.. Science.

[R2] Ferguson EL, Horvitz HR (1985). Identification and characterization of 22 genes that affect the vulval cell lineages of the nematode Caenorhabditis elegans.. Genetics.

[R3] Green JL, Inoue T, Sternberg PW (2008). Opposing Wnt pathways orient cell polarity during organogenesis.. Cell.

[R4] Minor PJ, He TF, Sohn CH, Asthagiri AR, Sternberg PW (2013). FGF signaling regulates Wnt ligand expression to control vulval cell lineage polarity in C. elegans.. Development.

[R5] Sulston JE, Horvitz HR (1977). Post-embryonic cell lineages of the nematode, Caenorhabditis elegans.. Dev Biol.

[R6] Whangbo J, Harris J, Kenyon C (2000). Multiple levels of regulation specify the polarity of an asymmetric cell division in C. elegans.. Development.

